# Rapid and Effective Electrical Conductivity Improvement of the Ag NW-Based Conductor by Using the Laser-Induced Nano-Welding Process

**DOI:** 10.3390/mi8050164

**Published:** 2017-05-19

**Authors:** Phillip Lee, Jinhyeong Kwon, Jinhwan Lee, Habeom Lee, Young D. Suh, Sukjoon Hong, Junyeob Yeo

**Affiliations:** 1 Photo-Electronic Hybrids Research Center, National Agenda Research Division, Korea Institute of Science and Technology (KIST), 5 Hwarang-ro 14-gil, Seongbuk-gu, Seoul 02792, Korea; phillip@kist.re.kr; 2 Applied Nano and Thermal Science (ANTS) Lab, Department of Mechanical Engineering, Seoul National University, 1 Gwanak-ro, Gwanak-gu, Seoul 00826, Korea; jhs0909k@snu.ac.kr (J.K.); mir.ljh@gmail.com (J.L.); habeom.lee@snu.ac.kr (H.L.); youngduksuh@gmail.com (Y.D.S.); 3 Department of Mechanical Engineering, Hanyang University, 55 Hanyangdaehak-ro, Sangnok-gu, Ansan, Gyeonggi-do 15588, Korea; 4 Novel Applied Nano Optics (NANO) Lab, Department of Physics, Kyungpook National University, 80 Daehak-ro, Bukgu, Daegu 41566, Korea

**Keywords:** laser, laser-induced nano-welding, pulse laser, silver nanowires, silver nanowire percolation networks, transparent flexible conductor

## Abstract

To date, the silver nanowire-based conductor has been widely used for flexible/stretchable electronics due to its several advantages. The optical nanowire annealing process has also received interest as an alternative annealing process to the Ag nanowire (NW)-based conductor. In this study, we present an analytical investigation on the phenomena of the Ag NWs’ junction and welding properties under laser exposure. The two different laser-induced welding processes (nanosecond (ns) pulse laser-induced nano-welding (LINW) and continuous wave (cw) scanning LINW) are applied to the Ag NW percolation networks. The Ag NWs are selectively melted and merged at the junction of Ag NWs under very short laser exposure; these results are confirmed by scanning electron microscope (SEM), focused-ion beam (FIB), electrical measurement, and finite difference time domain (FDTD) simulation.

## 1. Introduction

For almost two decades, the flexible transparent conductor and stretchable conductor have received huge interest from many researchers and industry professionals. Since flexible electronics and stretchable sensors are core devices in wearable computer systems, as a next generation electronics platform, manufacturing/fabrication process technologies as well as flexible electronics compatible materials will be more important in the future.

Meanwhile, among the materials for the transparent conductor, fluorine-doped tin oxide (FTO) thin film is the most popular and famous material in research and industry fields. However, FTO thin film is not an appropriate material for the flexible transparent conductor since it is usually delicate and brittle. Hence, instead of FTO thin film, alternative conducting nanomaterials such as carbon nanotube (CNT) [1,2], graphene [3,4,5], metal nanoparticle (NP) mesh [6,7,8,9], metal nanowires (NWs) [10,11,12,13,14,15,16,17,18,19], and metal nano-thin film [20,21] are used in the research field for the flexible transparent conductor and stretchable conductor. 

Among the nanomaterials for flexible electronics, silver (Ag) NWs have been widely used as a flexible transparent conductor [10,11,12,13,16,22,23,24] and stretchable conductor [15,17,25] due to various advantages such as high electrical conductivity, high transparency, high ductility and simple fabrication process methods. However, a post thermal annealing process is usually required to increase electrical conductivity since Ag NWs are usually too short (up to ~100 μm) to cover the wide area and are generally covered by capping polymer such as polyvinylpyrrolidone (PVP) which hinders electrical conductivity. The conventional thermal annealing process, such as a hot plate and convection oven, is simple and easily applicable to the Ag NW percolation networks for electrical conductivity improvement, while the thermal annealing process has disadvantages such as oxidation problems and long processing time. In particular, the thermal annealing process is not suitable to the flexible polymer substrate due to the low melting temperature of the polymer. Thus, it is important to conduct a post thermal annealing process with low temperature (below 200 °C) to prevent the damages of the flexible polymer substrate.

Recently, the optical NWs annealing process [14,15,18,19,26,27,28] was introduced to anneal metal NW percolation networks for the improvement of electrical conductivity. Compared to the conventional thermal annealing process, since optical energy in the optical NWs annealing process is irradiated to the sample over a very short time by a ultraviolet (UV) lamp [14], flash light [26] or scanning laser [18], the optical NWs annealing process is very rapid and suitable for the flexible polymer substrate without any macroscopic damages or deformation of the substrate [29,30] under ambient conditions. In addition, oxidation problems on metal NWs during processing are suppressed due to fast processing [18].

Most previous research [15,18,19,26,27,31] focuses on the fabrication of flexible/stretchable electronics and their applications. However, in this study, we attempt to examine mainly the phenomena of Ag NWs’ junction and welding properties when a laser is irradiated to the Ag NW percolation networks. In particular, we focus on how the local laser exposure time (from ns to μs) affects the Ag NWs’ percolation networks in laser processing. Thus, we compare two different laser-induced nano-welding (LINW) processes as a post annealing process of Ag NWs: the continuous wave (cw) laser scanning system and the nanosecond (ns) pulse laser system. Although the processing mechanism of two LINW processes is basically the same in terms of using laser energy, there are several similar and different results, which are mentioned in the text.

## 2. Methods

### 2.1. Experimental Procedure

**Figure 1:**
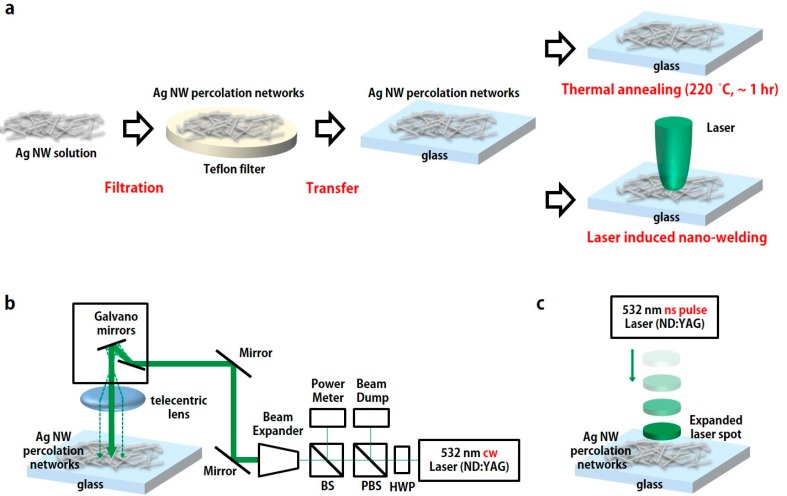
Schematic diagram of the thermal annealing process and laser-induced nano-welding process for Ag nanowires (NWs) percolation networks. (**a**) Sample preparation flow chart. Firstly, Ag NWs are synthesized in a solution by polyol synthesis. Afterwards, they are filtered on the Teflon filter and transferred onto the glass substrate to form Ag NW percolation networks on glass substrate. Finally, the thermal annealing process or laser-induced welding process is applied to Ag NW percolation networks for the improvement of electrical conductivity. The laser-induced nano-welding process through (**b**) the continuous wave (cw) laser scanning system and (**c**) the ns pulse laser system.

Figure 1a shows the preparation of an Ag NW-based conductor sample. In order to prepare a film of Ag NW percolation networks on the substrate, Ag NWs are deposited according to the following procedures. Firstly, Ag NWs are synthesized in a solution via the polyol synthesis method [32]. Afterwards, synthesized Ag NWs are filtered out onto the Teflon filter and transferred onto the glass substrate successively. The diameter of the transferred Ag NW percolation networks is ~36 mm due to the size of the Teflon filter. Since transferred Ag NW percolation networks on the glass substrate are weakly bound, the post thermal annealing process, such as a hot plate and furnace, is required to increase the electrical conductivity of the Ag NW percolation networks. Additionally, the LINW process is conducted to the Ag NW percolation networks as an alternative post annealing process for comparison.

### 2.2. Optical Setup

As we mentioned, two different LINW processes (the cw laser scanning system and the ns pulse laser system) are compared in this study. Figure 1b shows the cw laser scanning system which is combined with Galvano scanning mirrors and a telecentric lens [30,33]. In the cw laser scanning system, a 532 nm cw laser (Millennia 5W, Spectra Physics, Santa Clara, CA, USA) is used on the sample. As shown in Figure 1b, the power of the emitted laser beam is easily controlled through the half wave plate (HWP) and polarized beam splitter (PBS). The beam expander is placed afterwards to enlarge the laser beam for a flat wavefront of laser beam. The angle of the laser beam is deviated by a laser scanner (HurryScan II, Scanlab, Puchheim, Germany) which consists of two electrically driven Galvano mirrors. Afterwards, the laser beam is uniformly focused (10 μm) on the 2D focal plane, without distortion aberration, by a long focal distance f-theta lens (*f* = 103 mm). The prepared sample that consists of Ag NW percolation networks is placed on the focal plane of the f-theta lens. Figure 1c shows the ns pulse laser system which consists of a 532 nm ns pulse laser (Tempest 300, NewWave, Redwood City, CA, USA) and beam expander. The pulse duration and the repetition rate of the ns laser are 5 ns and 10 Hz, respectively. Since the energy of the applied ns pulse laser is extremely high, only one single shot with proper energy density (mJ/cm^2^) is enough for the enhancement of electrical conductivity in Ag NW percolation networks. However, excessive laser energy can ablate Ag NW percolation networks, thus the energy density in the ns pulse laser and the power density (W/cm^2^) in the cw laser are carefully controlled by adjustment of the beam waist area through the beam expander and power adjustment, respectively.

### 2.3. Laser Processing

Firstly, the prepared sample is placed at the focal plane of the applied laser. Afterwards, the laser is irradiated on the sample. In the cw scanning LINW process, the diameter of the prepared sample is ~36 mm. Additionally, the spot size of the focused laser, the laser scanning speed, and the pitch of scanning are 10 μm, 100 mm/s, and 10 μm, respectively. Since the total processing time in the cw scanning LINW process is dependent on sample size and laser scanning speed, fast laser scanning speed is desirable to reduce the processing time. Meanwhile, the laser dwell time, τ (τ = 2*W*_0_/*v*; τ, *W*_0_, and *v* are dwell time, beam waist, and scanning speed, respectively), is decreased as the scanning speed increases. Thus, laser energy density (power density by dwell time) and total processing time are in a trade-off relationship, and a laser scanning speed of 100 mm/s is chosen as an optimum scanning speed in this study.

In the case of the ns pulse LINW process, the laser beam is expanded by the beam expander to reduce laser energy density (energy per unit area), since the pulse laser energy is sufficiently high (see Figure 1c). The prepared sample size of Ag NW percolation networks is 6 mm by 6 mm in the ns pulse LINW process, and the extended laser beam illuminates and covers the entire prepared sample area (see Figure S1 in the Supplementary Information).

## 3. Results and Discussion

**Figure 2:**
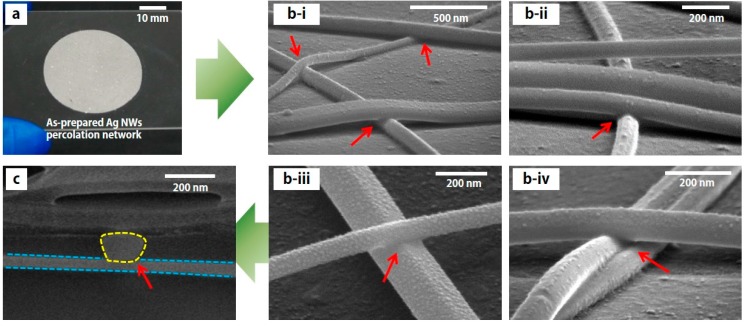
(**a**) Photographic image of the Ag NW percolation networks on the glass substrate (30% transmittance). (**b**) Magnified scanning electron microscope (SEM) images of the Ag NW percolation networks after the laser-induced welding process. (**c**) Cross-sectional SEM image of the junction of the Ag NW percolation networks after the laser-induced welding process. Two Ag NWs are melted and merged at the boundary of two Ag NWs (yellow dots and blue dots). Red arrows represent a junction of Ag NWs in (**b**,**c**).

Figure 2 shows a photographic image (Figure 2a) and scanning electron microscope (SEM) images (Figure 2b,c) of Ag NW percolation networks after the LINW process. In the case of the conventional thermal annealing process, such as a hot plate [15] and convection oven, the thermal annealing process is often conducted at a low temperature (e.g., below 250 °C) on the flexible substrate due to the low melting temperature of the polymer substrate. Therefore, it is difficult to find meaningful differences in the SEM images of Ag NW percolation networks after the thermal annealing process compared to before the thermal annealing process at a low temperature (below 250 °C).

However, in the case of the LINW process (same for both ns pulse LINW and cw scanning LINW) under ambient conditions (room temperature and atmospheric pressure), melted and merged Ag NWs are found in SEM images after the LINW process, as shown in Figure 2b-i–2b-iv. Since the irradiated laser energy is intensively absorbed to heat up Ag NWs and the laser irradiation time is extremely short (from ns to μs), crossed Ag NWs are only melted and merged at the junction, without damaging the other area of Ag NWs, as shown in Figure 2b.

In order to verify the melting at the junction of Ag NWs, a cross-sectional SEM image at the boundary which is cut by a focused-ion beam (FIB), is examined. It is confirmed that the crossed area of two Ag NWs (yellow and blue dotted lines) are melted and fused at the junction, as shown in Figure 2c. The results for the flexible transparent conductor and stretchable conductor are very noticeable, since melted and merged Ag NW percolation networks will have better electrical/mechanical properties such as electrical conductivity, mechanical elongation, and mechanical strength [13,15,18,19].

**Figure 3:**
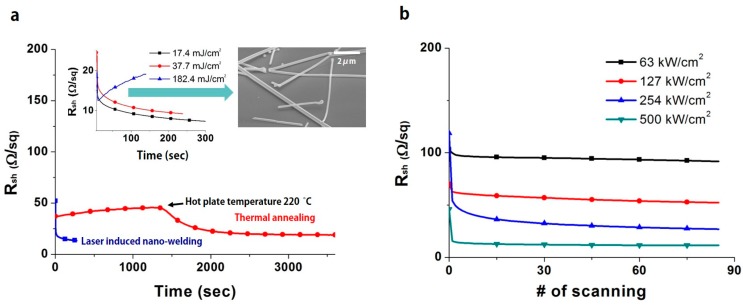
(**a**) Sheet resistance changes at various times in the thermal annealing process and ns pulse laser-induced nano-welding (LINW) process. The inset graph shows a magnified view of the ns pulse LINW process. SEM images show the laser ablation results of Ag NW percolation networks. (**b**) The sheet resistance changes with various numbers of laser scans and various laser power levels in the cw scanning LINW process.

Figure 3 shows sheet resistance changes at various times under the thermal annealing process and ns pulse LINW process. The transmittance of prepared Ag NW percolation networks in (a) and (b) are 95% and 96%, respectively. As shown in Figure 3a, the sheet resistance of Ag NW percolation networks is gradually increased and gently dropped below 20 Ω/sq with long processing time (over 1 h) under the thermal annealing process. At first, the sheet resistance is gradually increased due to oxidation formation and the resistance increase of Ag NWs to temperature change, according to their temperature coefficient of resistance [34,35]. Once the temperature of the hot plate reaches 220 °C (~1300 s), the sheet resistance starts to drop due to slight melting at the junction of Ag NW percolation networks. The thermal annealing process ensures stable low sheet resistance in Ag NW percolation networks and easily scales up the sample size. However, long processing time (over 1 h) is generally required to increase the electrical conductivity in the thermal annealing process, since a period of warm-up time for heating is required and only low temperature (below 250 °C) is available for the flexible substrate.

In contrast, the sheet resistance of Ag NW percolation networks drops rapidly in the ns pulse LINW process compared to the thermal annealing process. As shown in the inset graph of Figure 3a, the sheet resistance drops immediately after the start of pulse laser exposure with proper energy density (17.4 mJ/cm^2^ and 37.7 mJ/cm^2^). Even though a single pulse can be enough to improve the electrical conductivity of Ag NW percolation networks, continued laser pulses (10 Hz repetition rate) are employed in the sample to further improve the conductivity. However, extremely high laser energy density (182.4 mJ/cm^2^) can ablate/destroy Ag NW percolation networks (SEM image of Figure 3a), resulting in an increase of the sheet resistance of Ag NWs during the laser exposure—so-called “rebound”, as shown in the inset graph (light blue line) of Figure 3a. Thus, in Ag NW percolation networks in the ns pulse LINW process, moderate adjustment of the applied laser energy is required.

Similar to the ns pulse LINW process, in the case of the cw scanning LINW process, the sheet resistance of Ag NW percolation networks drops rapidly below 20 Ω/sq with high power density (500 kW/cm^2^), as shown in Figure 3b. The sheet resistance decreases slightly as the number of scans increases, while the sheet resistance drops considerably with high power density. The spot size (2*W*_0_) of the focused laser by the telecentric lens in laser scanning system is ~10 μm. Additionally, the dwell time is 10^−4^ s (10 μm/100 mms^−1^), thus it is also a very short time compared to the conventional thermal annealing process.

As a result, the LINW process is a more rapid and effective process (due to melting and merging) for improving the electrical conductivity of Ag NW percolation networks than the conventional thermal annealing process. Moreover, it is noticeable that there are no significant differences with respect to the different laser exposure time (from ns to μs) in laser processing. These results are confirmed by SEM images and electrical conductivity measurements. In addition, since the reaction in Ag NWs welding is conducted within an extremely short time—5 ns laser exposure time—this LINW process can be applied to the flexible substrate without any macroscopic damages or deformation of the substrate.

**Figure 4:**
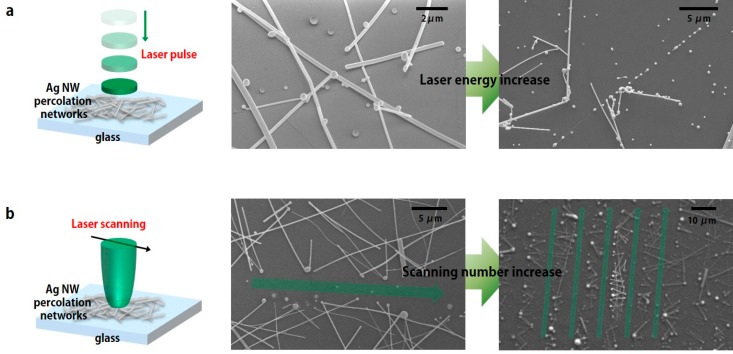
SEM images of Ag NW percolation networks when (**a**) the ns pulse LINW process and (**b**) the cw scanning LINW process are conducted under extremely high power/energy condition.

Figure 4 shows SEM images of ablated Ag NWs when the excessive laser energy is applied to the Ag NW percolation networks. Two different LINW processes show fairly different results at excessive laser energy density. Since an extended laser spot is applied to the Ag NW percolation networks in the ns pulse LINW process, entire Ag NWs are heated and melted, resulting in a queue of molten silver micro dots (right SEM images in Figure 4a). 

On the other hand, Ag NWs are selectively melted along the laser scanning direction in the cw scanning LINW process, as shown in Figure 4b (green arrows in SEM images). Since Ag NWs are ablated along the laser scanning direction, the remaining Ag NWs are locally connected to each other, thus this laser ablation technique is applied to fabricate patterned Ag NW mesh for flexible capacitive touch sensors [31].

**Figure 5:**
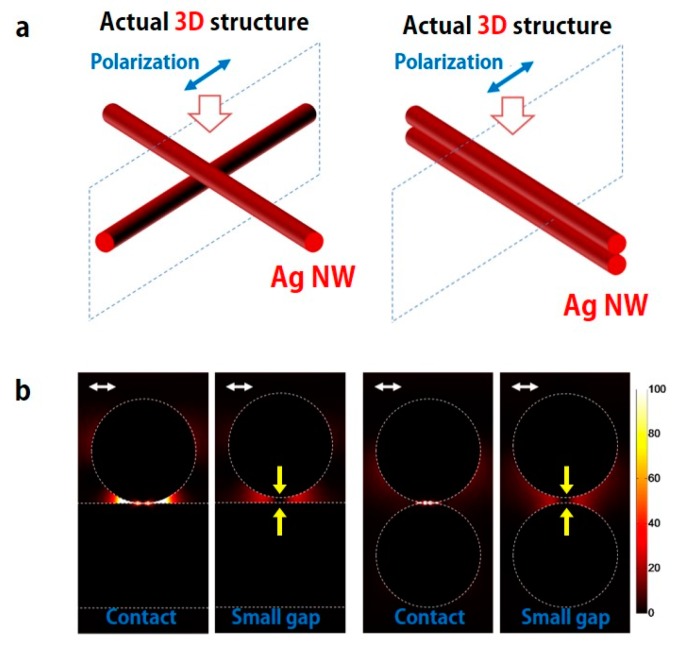
Finite difference time domain (FDTD) simulation at the junction of crossed Ag NWs. (**a**) Simulation layout of two crossed (left) and stacked (right) Ag NWs. (**b**) Electromagnetic field distribution at the junction with various conditions: crossed/contact, crossed/small gap, stacked/contact, stacked/small gap of two Ag NWs. The white arrow is the polarized direction of irradiated light. The yellow arrow indicates a small gap between two Ag NWs.

As shown in previous SEM images, Ag NWs are easily heated and melted in the LINW process when the proper laser energy is irradiated to the Ag NW percolation networks. It is well known that the electromagnetic field enhancements on the surface of Ag NW generate localized thermal heating due to surface plasmon polaritons (SPP) on the surface of Ag NWs [18,36,37,38,39]. This behavior can be seen in finite difference time domain (FDTD) simulation (Lumerical), as shown in Figure 5. In this simulation, transverse magnetic (TM) and transverse electric (TE) modes are considered for the two crossed and stacked Ag NWs. In order to simplify the simulation, the diameter size and shape of Ag NWs are fixed at 100 nm and circle, respectively. The complex permittivity of Ag is adopted from Palik, and a simulated pulse covers the wavelength range from 300 to 1000 nm. Total-field/scattering-field (TF-SF), together with perfectly matched layer (PML) formulation, has been employed.

As shown in Figure 5b, the electromagnetic field enhancements are extremely maximized at the junction of two crossed and stacked Ag NWs. In addition, electromagnetic field enhancement are still maximized near the junction of two Ag NWs, even though two Ag NWs are separated from each other by a small gap. The optical absorption, or the volumetric heat source density generated inside the metal, is calculated by [36]

(1) q(r→)=(ω/2)Im(ε)|E(r→)|2

As can be seen from the above equation, the optical absorption is directly proportional to the electrical field intensity, and the simulation result in Figure S2 shows that the optical absorption is concentrated at the regions where the field enhancement adjacent to the surface of the nanowire is the largest. This is the reason why Ag NWs are well melted and merged at the junction of Ag NWs, as shown in Figure 2. Additionally, these results are the reason why 5 nanoseconds is enough to improve the electrical conductivity of Ag NW percolation networks.

In summary, two different laser-induced nano-welding processes (ns pulse LINW and cw scanning LINW), as alternative post annealing processes, are investigated to enhance the electrical conductivity of an Ag NW-based conductor in this study. Thus, various phenomena of Ag NWs are examined when the laser irradiates to the Ag NW percolation networks. Through the various characterizations (SEM, FIB, and electrical measurements) and FDTD simulation, it is confirmed that there are no significant differences with respect to the different laser exposure time (from ns to μs). Additionally, the Ag NWs can be selectively melted and merged at the junction of Ag NWs within less than 5 nanoseconds laser exposure.

These results indicate that the LINW process is expected to apply to the flexible polymer substrate without any macroscopic damages or deformation of the substrate due to the rapid processing time and the effect of localized electromagnetic field enhancements. In addition, since melted and merged Ag NW percolation networks will have better electrical/mechanical properties, we expect that the LINW process will be applied to the fabrication of various flexible/stretchable electronics for better performance.

## Supplementary Materials

The following are available online at www.mdpi.com/2072-666X/8/5/164/s1. Figure S1: The sample preparation of Ag NWs percolation networks for the ns pulse (a) and cw scanning (b) LINW process. The transmittance of the sample is ~91%. Figure S2: Spatial profile of electrical field intensity and the corresponding optical power absorption.
