# Supplementary material for: Rapid and Effective Electrical Conductivity Improvement of the Ag NW-Based Conductor by Using the Laser-Induced Nano-Welding Process
Source: Micromachines (Basel). 2017 May 19;8(5):164. doi: 10.3390/mi8050164 (PMC6190447; doi:10.3390/mi8050164)
Supplement: Supplementary file 1 [file micromachines-08-00164-s001.pdf]

Supplementary Materials: Rapid and Effective Electrical Conductivity Improvement of the Ag NW-Based Conductor by Using the Laser-Induced Nano-Welding Process

Phillip Lee, Jinhyeong Kwon, Jinhwan Lee, Habeom Lee, Young D. Suh, Sukjoon Hong and Junyeob Yeo

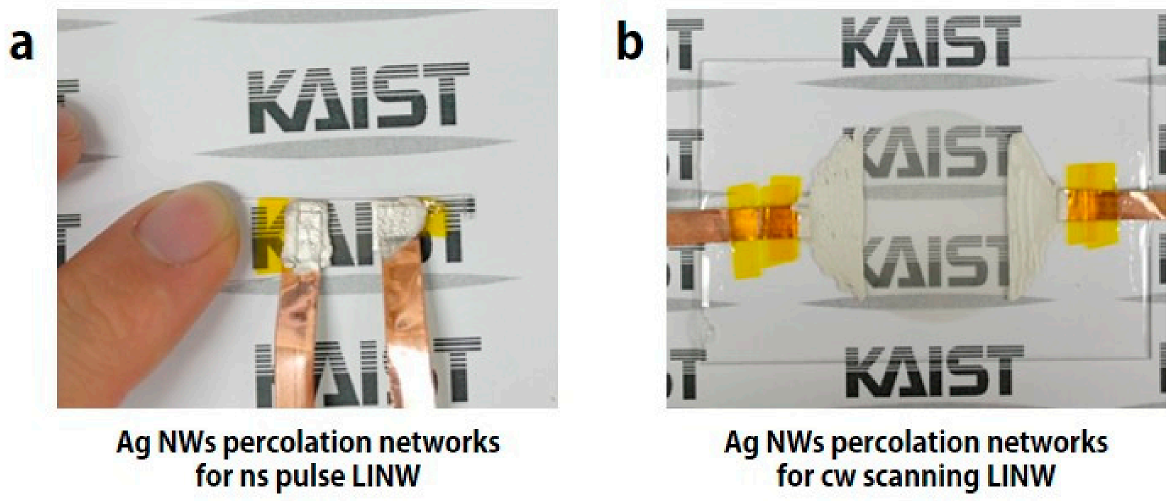

Figure S1. The sample preparation of Ag NWs percolation networks for the ns pulse (**a**) and cw scanning (**b**) LINW process. The transmittance of the sample is ~91 %.

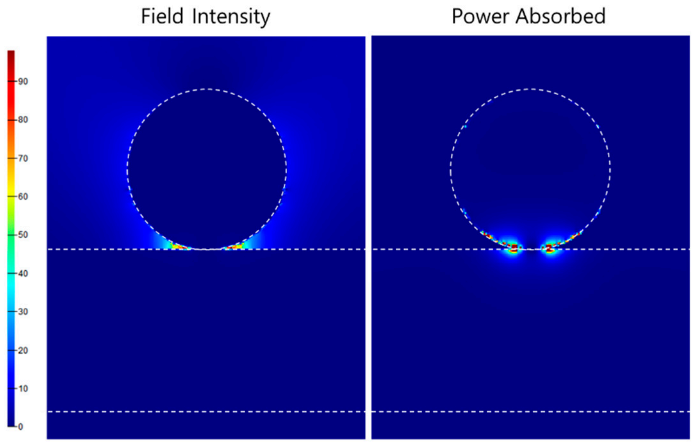

Figure S2. Spatial profile of electrical field intensity and the corresponding optical power absorption.
